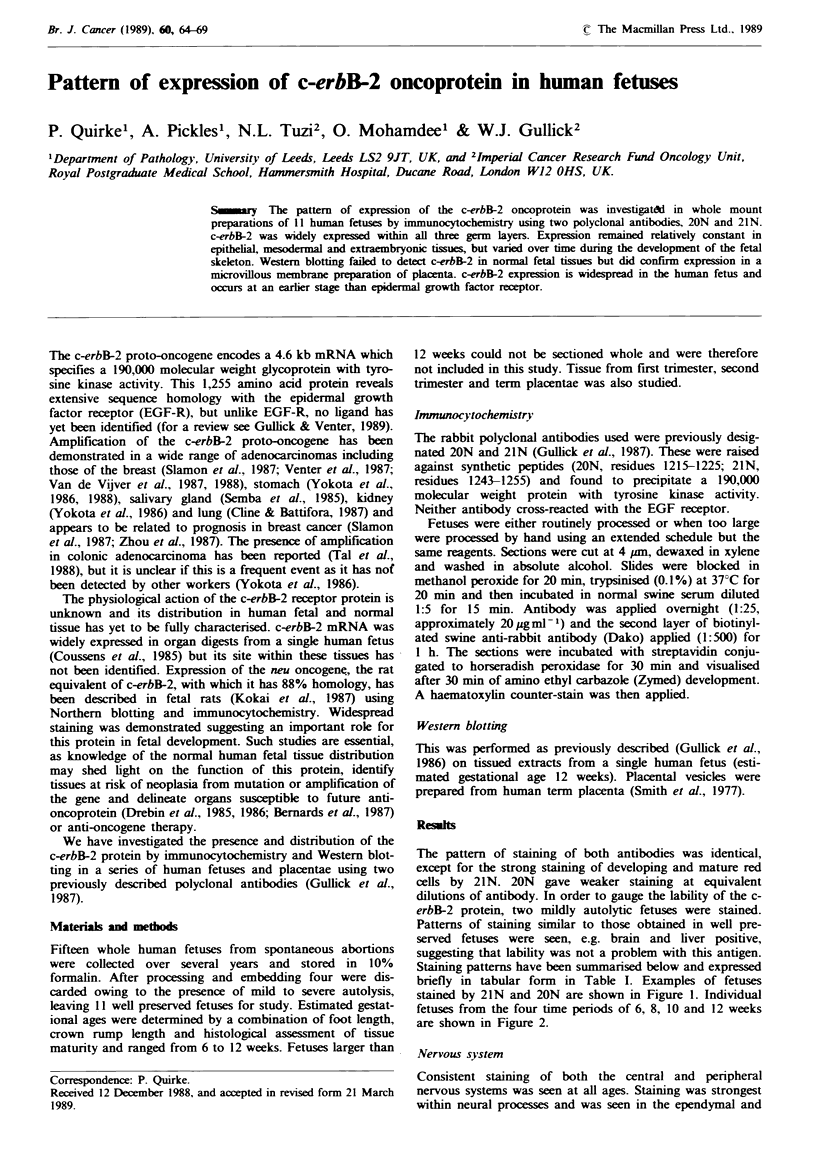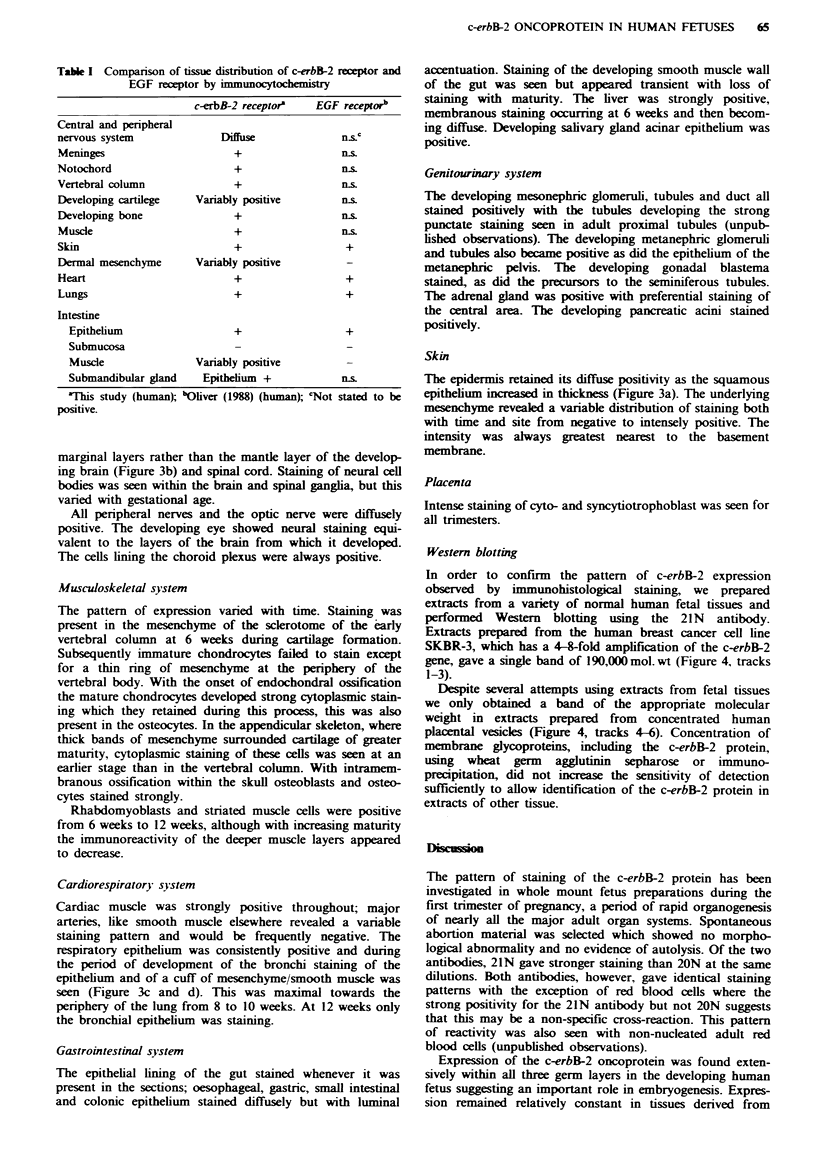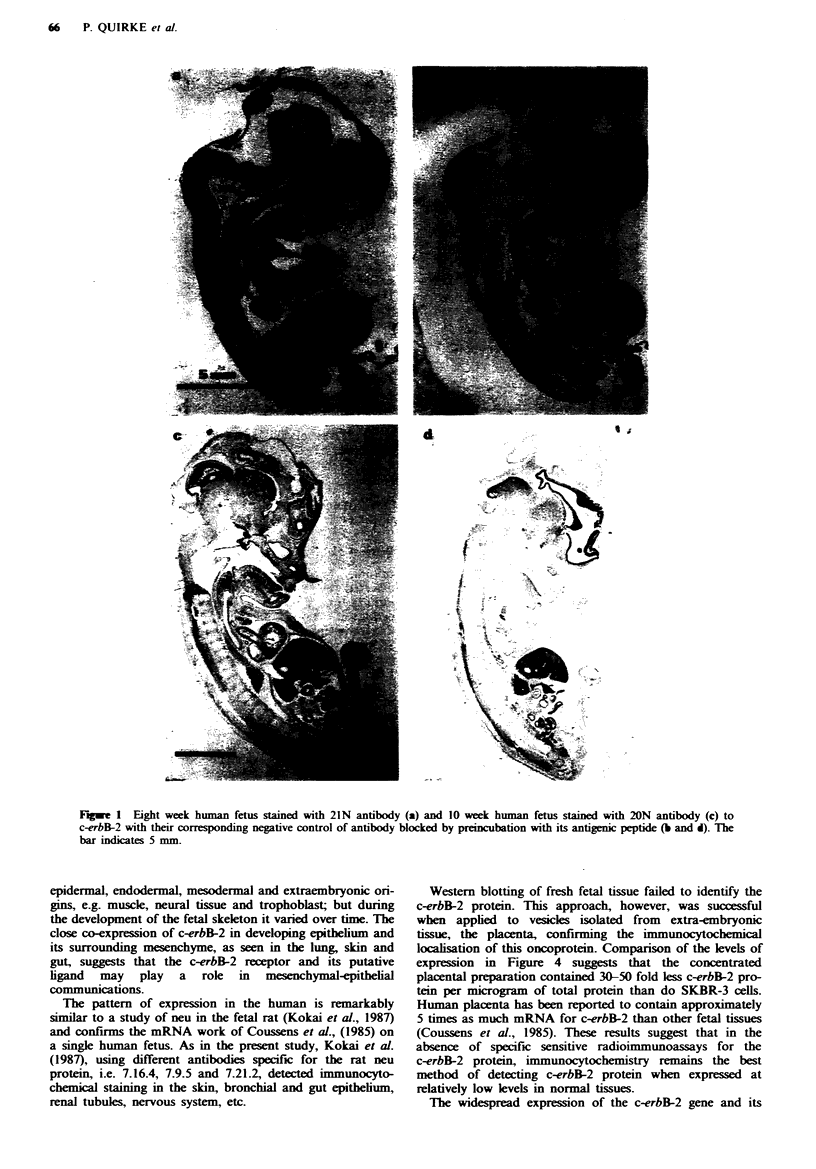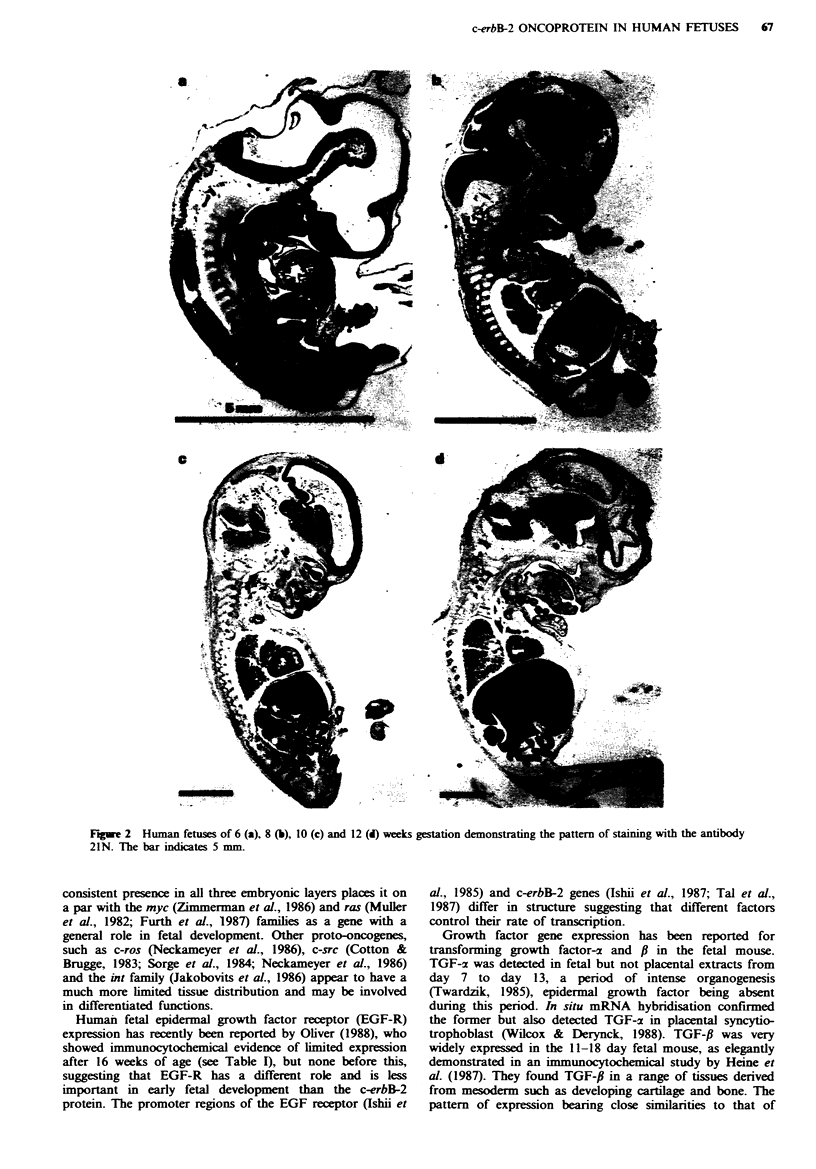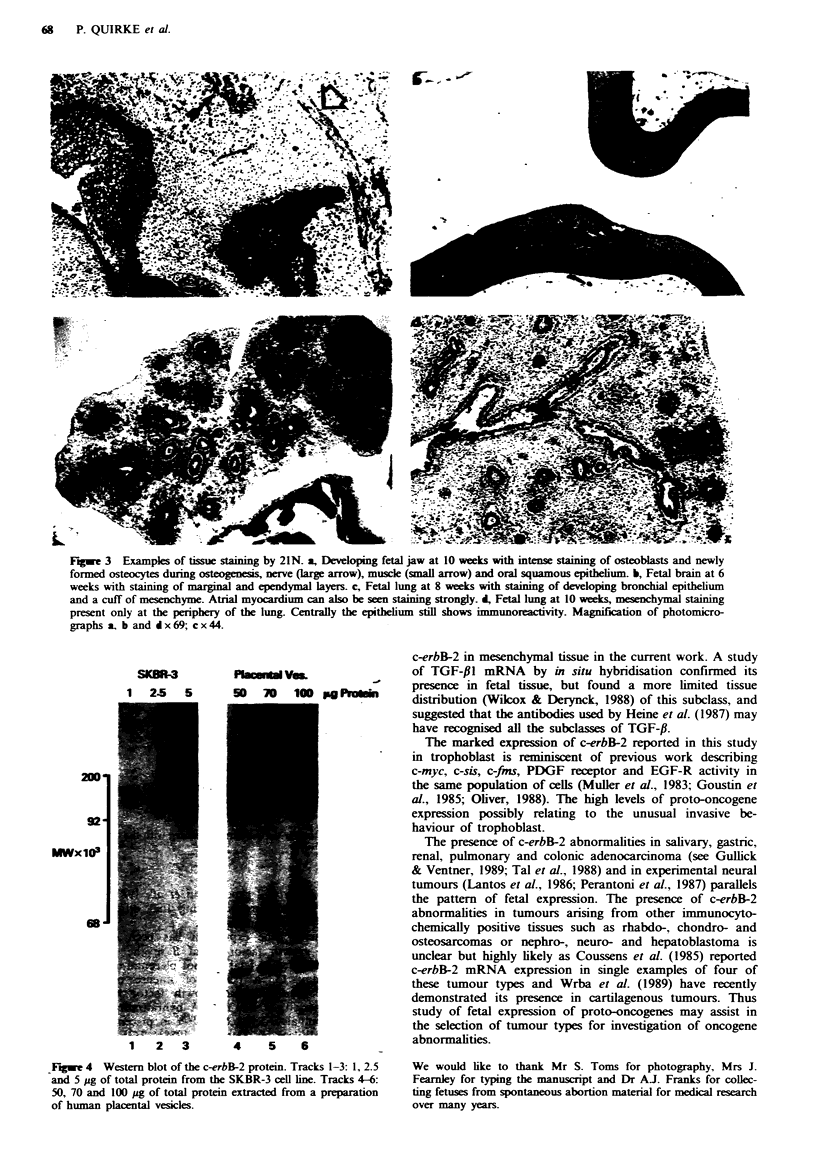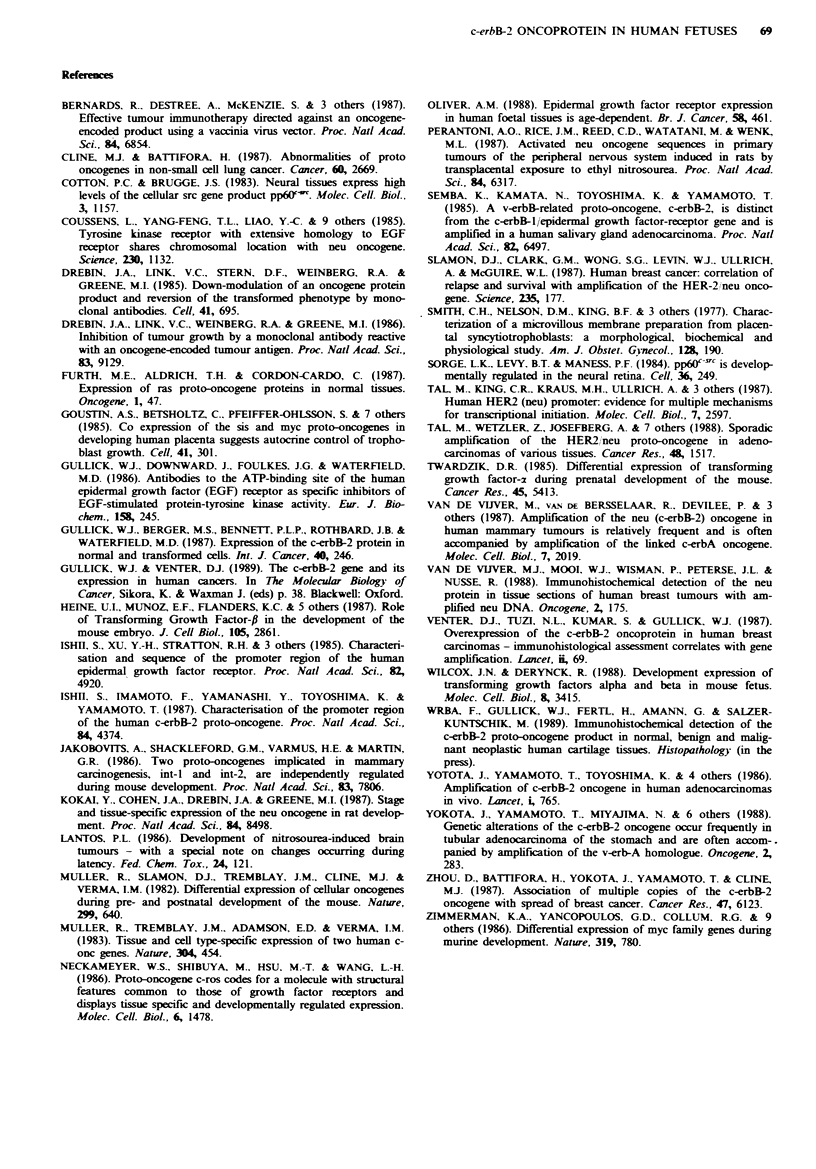# Pattern of expression of c-erbB-2 oncoprotein in human fetuses.

**DOI:** 10.1038/bjc.1989.221

**Published:** 1989-07

**Authors:** P. Quirke, A. Pickles, N. L. Tuzi, O. Mohamdee, W. J. Gullick

**Affiliations:** Department of Pathology, University of Leeds, UK.

## Abstract

**Images:**


Br. J. Cancer (1989), 60, 64–69 © The Macmillan Press Ltd., 1989

### Pattern of expression of c-*erb*B-2 oncoprotein in human fetuses

P. Quirke[1], A. Pickles[1], N.L. Tuzi[2], O. Mohamdee[1] & W.J. Gullick[2]

[1]*Department of Pathology, University of Leeds, Leeds LS2 9JT, UK, and* [2]*Imperial Cancer Research Fund Oncology Unit, Royal Postgraduate Medical School, Hammersmith Hospital, Ducane Road, London W12 0HS, UK.*

**Summary** The pattern of expression of the c-*erb*B-2 oncoprotein was investigated in whole mount preparations of 11 human fetuses by immunocytochemistry using two polyclonal antibodies, 20N and 21N. c-*erb*B-2 was widely expressed within all three germ layers. Expression remained relatively constant in epithelial, mesodermal and extraembryonic tissues, but varied over time during the development of the fetal skeleton. Western blotting failed to detect c-*erb*B-2 in normal fetal tissues but did confirm expression in a microvillous membrane preparation of placenta. c-*erb*B-2 expression is widespread in the human fetus and occurs at an earlier stage than epidermal growth factor receptor.

The c-*erb*B-2 proto-oncogene encodes a 4.6 kb mRNA which specifies a 190,000 molecular weight glycoprotein with tyrosine kinase activity. This 1,255 amino acid protein reveals extensive sequence homology with the epidermal growth factor receptor (EGF-R), but unlike EGF-R, no ligand has yet been identified (for a review see Gullick & Venter, 1989). Amplification of the c-*erb*B-2 proto-oncogene has been demonstrated in a wide range of adenocarcinomas including those of the breast (Slamon *et al.*, 1987; Venter *et al.*, 1987; Van de Vijver *et al.*, 1987, 1988), stomach (Yokota *et al.*, 1986, 1988), salivary gland (Semba *et al.*, 1985), kidney (Yokota *et al.*, 1986) and lung (Cline & Battifora, 1987) and appears to be related to prognosis in breast cancer (Slamon *et al.*, 1987; Zhou *et al.*, 1987). The presence of amplification in colonic adenocarcinoma has been reported (Tal *et al.*, 1988), but it is unclear if this is a frequent event as it has not been detected by other workers (Yokota *et al.*, 1986).

The physiological action of the c-*erb*B-2 receptor protein is unknown and its distribution in human fetal and normal tissue has yet to be fully characterised. c-*erb*B-2 mRNA was widely expressed in organ digests from a single human fetus (Coussens *et al.*, 1985) but its site within these tissues has not been identified. Expression of the *neu* oncogene, the rat equivalent of c-*erb*B-2, with which it has 88% homology, has been described in fetal rats (Kokai *et al.*, 1987) using Northern blotting and immunocytochemistry. Widespread staining was demonstrated suggesting an important role for this protein in fetal development. Such studies are essential, as knowledge of the normal human fetal tissue distribution may shed light on the function of this protein, identify tissues at risk of neoplasia from mutation or amplification of the gene and delineate organs susceptible to future anti-oncoprotein (Drebin *et al.*, 1985, 1986; Bernards *et al.*, 1987) or anti-oncogene therapy.

We have investigated the presence and distribution of the c-*erb*B-2 protein by immunocytochemistry and Western blotting in a series of human fetuses and placentae using two previously described polyclonal antibodies (Gullick *et al.*, 1987).

### Materials and methods

Fifteen whole human fetuses from spontaneous abortions were collected over several years and stored in 10% formalin. After processing and embedding four were discarded owing to the presence of mild to severe autolysis, leaving 11 well preserved fetuses for study. Estimated gestational ages were determined by a combination of foot length, crown rump length and histological assessment of tissue maturity and ranged from 6 to 12 weeks. Fetuses larger than 12 weeks could not be sectioned whole and were therefore not included in this study. Tissue from first trimester, second trimester and term placentae was also studied.

### *Immunocytochemistry*

The rabbit polyclonal antibodies used were previously designated 20N and 21N (Gullick *et al.*, 1987). These were raised against synthetic peptides (20N, residues 1215–1225; 21N, residues 1243–1255) and found to precipitate a 190,000 molecular weight protein with tyrosine kinase activity. Neither antibody cross-reacted with the EGF receptor.

Fetuses were either routinely processed or when too large were processed by hand using an extended schedule but the same reagents. Sections were cut at 4 $\mu$m, dewaxed in xylene and washed in absolute alcohol. Slides were blocked in methanol peroxide for 20 min, trypsinised (0.1%) at 37°C for 20 min and then incubated in normal swine serum diluted 1:5 for 15 min. Antibody was applied overnight (1:25, approximately 20 $\mu g\,ml^{-1}$) and the second layer of biotinylated swine anti-rabbit antibody (Dako) applied (1:500) for 1 h. The sections were incubated with streptavidin conjugated to horseradish peroxidase for 30 min and visualised after 30 min of amino ethyl carbazole (Zymed) development. A haematoxylin counter-stain was then applied.

### *Western blotting*

This was performed as previously described (Gullick *et al.*, 1986) on tissued extracts from a single human fetus (estimated gestational age 12 weeks). Placental vesicles were prepared from human term placenta (Smith *et al.*, 1977).

### Results

The pattern of staining of both antibodies was identical, except for the strong staining of developing and mature red cells by 21N. 20N gave weaker staining at equivalent dilutions of antibody. In order to gauge the lability of the c-*erb*B-2 protein, two mildly autolytic fetuses were stained. Patterns of staining similar to those obtained in well preserved fetuses were seen, e.g. brain and liver positive, suggesting that lability was not a problem with this antigen. Staining patterns have been summarised below and expressed briefly in tabular form in Table I. Examples of fetuses stained by 21N and 20N are shown in Figure 1. Individual fetuses from the four time periods of 6, 8, 10 and 12 weeks are shown in Figure 2.

### *Nervous system*

Consistent staining of both the central and peripheral nervous systems was seen at all ages. Staining was strongest within neural processes and was seen in the ependymal and

Correspondence: P. Quirke.
Received 12 December 1988, and accepted in revised form 21 March 1989.

**Table I** Comparison of tissue distribution of c-*erb*B-2 receptor and EGF receptor by immunocytochemistry

| | *c-erbB-2 receptor*[a] | *EGF receptor*[b] |
|---|---|---|
| Central and peripheral nervous system | Diffuse | n.s.[c] |
| Meninges | + | n.s. |
| Notochord | + | n.s. |
| Vertebral column | + | n.s. |
| Developing cartilege | Variably positive | n.s. |
| Developing bone | + | n.s. |
| Muscle | + | n.s. |
| Skin | + | + |
| Dermal mesenchyme | Variably positive | – |
| Heart | + | + |
| Lungs | + | + |
| Intestine | | |
| Epithelium | + | + |
| Submucosa | – | – |
| Muscle | Variably positive | – |
| Submandibular gland | Epithelium + | n.s. |

[a]This study (human); [b]Oliver (1988) (human); [c]Not stated to be positive.

marginal layers rather than the mantle layer of the developing brain (Figure 3b) and spinal cord. Staining of neural cell bodies was seen within the brain and spinal ganglia, but this varied with gestational age.

All peripheral nerves and the optic nerve were diffusely positive. The developing eye showed neural staining equivalent to the layers of the brain from which it developed. The cells lining the choroid plexus were always positive.

### *Musculoskeletal system*

The pattern of expression varied with time. Staining was present in the mesenchyme of the sclerotome of the early vertebral column at 6 weeks during cartilage formation. Subsequently immature chondrocytes failed to stain except for a thin ring of mesenchyme at the periphery of the vertebral body. With the onset of endochondral ossification the mature chondrocytes developed strong cytoplasmic staining which they retained during this process, this was also present in the osteocytes. In the appendicular skeleton, where thick bands of mesenchyme surrounded cartilage of greater maturity, cytoplasmic staining of these cells was seen at an earlier stage than in the vertebral column. With intramembranous ossification within the skull osteoblasts and osteocytes stained strongly.

Rhabdomyoblasts and striated muscle cells were positive from 6 weeks to 12 weeks, although with increasing maturity the immunoreactivity of the deeper muscle layers appeared to decrease.

### *Cardiorespiratory system*

Cardiac muscle was strongly positive throughout; major arteries, like smooth muscle elsewhere revealed a variable staining pattern and would be frequently negative. The respiratory epithelium was consistently positive and during the period of development of the bronchi staining of the epithelium and of a cuff of mesenchyme/smooth muscle was seen (Figure 3c and d). This was maximal towards the periphery of the lung from 8 to 10 weeks. At 12 weeks only the bronchial epithelium was staining.

### *Gastrointestinal system*

The epithelial lining of the gut stained whenever it was present in the sections; oesophageal, gastric, small intestinal and colonic epithelium stained diffusely but with luminal accentuation. Staining of the developing smooth muscle wall of the gut was seen but appeared transient with loss of staining with maturity. The liver was strongly positive, membranous staining occurring at 6 weeks and then becoming diffuse. Developing salivary gland acinar epithelium was positive.

### *Genitourinary system*

The developing mesonephric glomeruli, tubules and duct all stained positively with the tubules developing the strong punctate staining seen in adult proximal tubules (unpublished observations). The developing metanephric glomeruli and tubules also became positive as did the epithelium of the metanephric pelvis. The developing gonadal blastema stained, as did the precursors to the seminiferous tubules. The adrenal gland was positive with preferential staining of the central area. The developing pancreatic acini stained positively.

### *Skin*

The epidermis retained its diffuse positivity as the squamous epithelium increased in thickness (Figure 3a). The underlying mesenchyme revealed a variable distribution of staining both with time and site from negative to intensely positive. The intensity was always greatest nearest to the basement membrane.

### *Placenta*

Intense staining of cyto- and syncytiotrophoblast was seen for all trimesters.

### *Western blotting*

In order to confirm the pattern of c-*erb*B-2 expression observed by immunohistological staining, we prepared extracts from a variety of normal human fetal tissues and performed Western blotting using the 21N antibody. Extracts prepared from the human breast cancer cell line SKBR-3, which has a 4–8-fold amplification of the c-*erb*B-2 gene, gave a single band of 190,000 mol. wt (Figure 4, tracks 1–3).

Despite several attempts using extracts from fetal tissues we only obtained a band of the appropriate molecular weight in extracts prepared from concentrated human placental vesicles (Figure 4, tracks 4–6). Concentration of membrane glycoproteins, including the c-*erb*B-2 protein, using wheat germ agglutinin sepharose or immunoprecipitation, did not increase the sensitivity of detection sufficiently to allow identification of the c-*erb*B-2 protein in extracts of other tissue.

### Discussion

The pattern of staining of the c-*erb*B-2 protein has been investigated in whole mount fetus preparations during the first trimester of pregnancy, a period of rapid organogenesis of nearly all the major adult organ systems. Spontaneous abortion material was selected which showed no morphological abnormality and no evidence of autolysis. Of the two antibodies, 21N gave stronger staining than 20N at the same dilutions. Both antibodies, however, gave identical staining patterns with the exception of red blood cells where the strong positivity for the 21N antibody but not 20N suggests that this may be a non-specific cross-reaction. This pattern of reactivity was also seen with non-nucleated adult red blood cells (unpublished observations).

Expression of the c-*erb*B-2 oncoprotein was found extensively within all three germ layers in the developing human fetus suggesting an important role in embryogenesis. Expression remained relatively constant in tissues derived from

**Figure 1** Eight week human fetus stained with 21N antibody (**a**) and 10 week human fetus stained with 20N antibody (**c**) to c-*erb*B-2 with their corresponding negative control of antibody blocked by preincubation with its antigenic peptide (**b** and **d**). The bar indicates 5 mm.

epidermal, endodermal, mesodermal and extraembryonic origins, e.g. muscle, neural tissue and trophoblast; but during the development of the fetal skeleton it varied over time. The close co-expression of c-*erb*B-2 in developing epithelium and its surrounding mesenchyme, as seen in the lung, skin and gut, suggests that the c-*erb*B-2 receptor and its putative ligand may play a role in mesenchymal-epithelial communications.

The pattern of expression in the human is remarkably similar to a study of neu in the fetal rat (Kokai *et al.*, 1987) and confirms the mRNA work of Coussens *et al.*, (1985) on a single human fetus. As in the present study, Kokai *et al.* (1987), using different antibodies specific for the rat neu protein, i.e. 7.16.4, 7.9.5 and 7.21.2, detected immunocytochemical staining in the skin, bronchial and gut epithelium, renal tubules, nervous system, etc.

Western blotting of fresh fetal tissue failed to identify the c-*erb*B-2 protein. This approach, however, was successful when applied to vesicles isolated from extra-embryonic tissue, the placenta, confirming the immunocytochemical localisation of this oncoprotein. Comparison of the levels of expression in Figure 4 suggests that the concentrated placental preparation contained 30–50 fold less c-*erb*B-2 protein per microgram of total protein than do SKBR-3 cells. Human placenta has been reported to contain approximately 5 times as much mRNA for c-*erb*B-2 than other fetal tissues (Coussens *et al.*, 1985). These results suggest that in the absence of specific sensitive radioimmunoassays for the c-*erb*B-2 protein, immunocytochemistry remains the best method of detecting c-*erb*B-2 protein when expressed at relatively low levels in normal tissues.

The widespread expression of the c-*erb*B-2 gene and its

**Figure 2** Human fetuses of 6 (**a**), 8 (**b**), 10 (**c**) and 12 (**d**) weeks gestation demonstrating the pattern of staining with the antibody 21N. The bar indicates 5 mm.

consistent presence in all three embryonic layers places it on a par with the *myc* (Zimmerman *et al.*, 1986) and *ras* (Muller *et al.*, 1982; Furth *et al.*, 1987) families as a gene with a general role in fetal development. Other proto-oncogenes, such as c-*ros* (Neckameyer *et al.*, 1986), c-*src* (Cotton & Brugge, 1983; Sorge *et al.*, 1984; Neckameyer *et al.*, 1986) and the *int* family (Jakobovits *et al.*, 1986) appear to have a much more limited tissue distribution and may be involved in differentiated functions.

Human fetal epidermal growth factor receptor (EGF-R) expression has recently been reported by Oliver (1988), who showed immunocytochemical evidence of limited expression after 16 weeks of age (see Table I), but none before this, suggesting that EGF-R has a different role and is less important in early fetal development than the c-*erb*B-2 protein. The promoter regions of the EGF receptor (Ishii *et al.*, 1985) and c-*erb*B-2 genes (Ishii *et al.*, 1987; Tal *et al.*, 1987) differ in structure suggesting that different factors control their rate of transcription.

Growth factor gene expression has been reported for transforming growth factor-α and β in the fetal mouse. TGF-α was detected in fetal but not placental extracts from day 7 to day 13, a period of intense organogenesis (Twardzik, 1985), epidermal growth factor being absent during this period. *In situ* mRNA hybridisation confirmed the former but also detected TGF-α in placental syncytiotrophoblast (Wilcox & Derynck, 1988). TGF-β was very widely expressed in the 11–18 day fetal mouse, as elegantly demonstrated in an immunocytochemical study by Heine *et al.* (1987). They found TGF-β in a range of tissues derived from mesoderm such as developing cartilage and bone. The pattern of expression bearing close similarities to that of

**Figure 3** Examples of tissue staining by 21N. **a**, Developing fetal jaw at 10 weeks with intense staining of osteoblasts and newly formed osteocytes during osteogenesis, nerve (large arrow), muscle (small arrow) and oral squamous epithelium. **b**, Fetal brain at 6 weeks with staining of marginal and ependymal layers. **c**, Fetal lung at 8 weeks with staining of developing bronchial epithelium and a cuff of mesenchyme. Atrial myocardium can also be seen staining strongly. **d**, Fetal lung at 10 weeks, mesenchymal staining present only at the periphery of the lung. Centrally the epithelium still shows immunoreactivity. Magnification of photomicrographs **a**, **b** and **d** ×69; **c** ×44.

**Figure 4** Western blot of the c-*erb*B-2 protein. Tracks 1–3: 1, 2.5 and 5 µg of total protein from the SKBR-3 cell line. Tracks 4–6: 50, 70 and 100 µg of total protein extracted from a preparation of human placental vesicles.

c-*erb*B-2 in mesenchymal tissue in the current work. A study of TGF-β1 mRNA by *in situ* hybridisation confirmed its presence in fetal tissue, but found a more limited tissue distribution (Wilcox & Derynck, 1988) of this subclass, and suggested that the antibodies used by Heine *et al.* (1987) may have recognised all the subclasses of TGF-β.

The marked expression of c-*erb*B-2 reported in this study in trophoblast is reminiscent of previous work describing c-*myc*, c-*sis*, c-*fms*, PDGF receptor and EGF-R activity in the same population of cells (Muller *et al.*, 1983; Goustin *et al.*, 1985; Oliver, 1988). The high levels of proto-oncogene expression possibly relating to the unusual invasive behaviour of trophoblast.

The presence of c-*erb*B-2 abnormalities in salivary, gastric, renal, pulmonary and colonic adenocarcinoma (see Gullick & Ventner, 1989; Tal *et al.*, 1988) and in experimental neural tumours (Lantos *et al.*, 1986; Perantoni *et al.*, 1987) parallels the pattern of fetal expression. The presence of c-*erb*B-2 abnormalities in tumours arising from other immunocytochemically positive tissues such as rhabdo-, chondro- and osteosarcomas or nephro-, neuro- and hepatoblastoma is unclear but highly likely as Coussens *et al.* (1985) reported c-*erb*B-2 mRNA expression in single examples of four of these tumour types and Wrba *et al.* (1989) have recently demonstrated its presence in cartilagenous tumours. Thus study of fetal expression of proto-oncogenes may assist in the selection of tumour types for investigation of oncogene abnormalities.

We would like to thank Mr S. Toms for photography, Mrs J. Fearnley for typing the manuscript and Dr A.J. Franks for collecting fetuses from spontaneous abortion material for medical research over many years.

**References**

BERNARDS, R., DESTREE, A., McKENZIE, S. & 3 others (1987). Effective tumour immunotherapy directed against an oncogene-encoded product using a vaccinia virus vector. *Proc. Natl Acad. Sci.*, **84**, 6854.

CLINE, M.J. & BATTIFORA, H. (1987). Abnormalities of proto oncogenes in non-small cell lung cancer. *Cancer*, **60**, 2669.

COTTON, P.C. & BRUGGE, J.S. (1983). Neural tissues express high levels of the cellular src gene product pp60$^{c\text{-}src}$. *Molec. Cell. Biol.*, **3**, 1157.

COUSSENS, L., YANG-FENG, T.L., LIAO, Y.-C. & 9 others (1985). Tyrosine kinase receptor with extensive homology to EGF receptor shares chromosomal location with neu oncogene. *Science*, **230**, 1132.

DREBIN, J.A., LINK, V.C., STERN, D.F., WEINBERG, R.A. & GREENE, M.I. (1985). Down-modulation of an oncogene protein product and reversion of the transformed phenotype by monoclonal antibodies. *Cell*, **41**, 695.

DREBIN, J.A., LINK, V.C., WEINBERG, R.A. & GREENE, M.I. (1986). Inhibition of tumour growth by a monoclonal antibody reactive with an oncogene-encoded tumour antigen. *Proc. Natl Acad. Sci.*, **83**, 9129.

FURTH, M.E., ALDRICH, T.H. & CORDON-CARDO, C. (1987). Expression of ras proto-oncogene proteins in normal tissues. *Oncogene*, **1**, 47.

GOUSTIN, A.S., BETSHOLTZ, C., PFEIFFER-OHLSSON, S. & 7 others (1985). Co expression of the sis and myc proto-oncogenes in developing human placenta suggests autocrine control of trophoblast growth. *Cell*, **41**, 301.

GULLICK, W.J., DOWNWARD, J., FOULKES, J.G. & WATERFIELD, M.D. (1986). Antibodies to the ATP-binding site of the human epidermal growth factor (EGF) receptor as specific inhibitors of EGF-stimulated protein-tyrosine kinase activity. *Eur. J. Biochem.*, **158**, 245.

GULLICK, W.J., BERGER, M.S., BENNETT, P.L.P., ROTHBARD, J.B. & WATERFIELD, M.D. (1987). Expression of the c-erbB-2 protein in normal and transformed cells. *Int. J. Cancer*, **40**, 246.

GULLICK, W.J. & VENTER, D.J. (1989). The c-erbB-2 gene and its expression in human cancers. In *The Molecular Biology of Cancer*, Sikora, K. & Waxman J. (eds) p. 38. Blackwell: Oxford.

HEINE, U.I., MUNOZ, E.F., FLANDERS, K.C. & 5 others (1987). Role of Transforming Growth Factor-$\beta$ in the development of the mouse embryo. *J. Cell Biol.*, **105**, 2861.

ISHII, S., XU, Y.-H., STRATTON, R.H. & 3 others (1985). Characterisation and sequence of the promoter region of the human epidermal growth factor receptor. *Proc. Natl Acad. Sci.*, **82**, 4920.

ISHII, S., IMAMOTO, F., YAMANASHI, Y., TOYOSHIMA, K. & YAMAMOTO, T. (1987). Characterisation of the promoter region of the human c-erbB-2 proto-oncogene. *Proc. Natl Acad. Sci.*, **84**, 4374.

JAKOBOVITS, A., SHACKLEFORD, G.M., VARMUS, H.E. & MARTIN, G.R. (1986). Two proto-oncogenes implicated in mammary carcinogenesis, int-1 and int-2, are independently regulated during mouse development. *Proc. Natl Acad. Sci.*, **83**, 7806.

KOKAI, Y., COHEN, J.A., DREBIN, J.A. & GREENE, M.I. (1987). Stage and tissue-specific expression of the neu oncogene in rat development. *Proc. Natl Acad. Sci.*, **84**, 8498.

LANTOS, P.L. (1986). Development of nitrosourea-induced brain tumours – with a special note on changes occurring during latency. *Fed. Chem. Tox.*, **24**, 121.

MULLER, R., SLAMON, D.J., TREMBLAY, J.M., CLINE, M.J. & VERMA, I.M. (1982). Differential expression of cellular oncogenes during pre- and postnatal development of the mouse. *Nature*, **299**, 640.

MULLER, R., TREMBLAY, J.M., ADAMSON, E.D. & VERMA, I.M. (1983). Tissue and cell type-specific expression of two human c-onc genes. *Nature*, **304**, 454.

NECKAMEYER, W.S., SHIBUYA, M., HSU, M.-T. & WANG, L.-H. (1986). Proto-oncogene c-ros codes for a molecule with structural features common to those of growth factor receptors and displays tissue specific and developmentally regulated expression. *Molec. Cell. Biol.*, **6**, 1478.

OLIVER, A.M. (1988). Epidermal growth factor receptor expression in human foetal tissues is age-dependent. *Br. J. Cancer*, **58**, 461.

PERANTONI, A.O., RICE, J.M., REED, C.D., WATATANI, M. & WENK, M.L. (1987). Activated neu oncogene sequences in primary tumours of the peripheral nervous system induced in rats by transplacental exposure to ethyl nitrosourea. *Proc. Natl Acad. Sci.*, **84**, 6317.

SEMBA, K., KAMATA, N., TOYOSHIMA, K. & YAMAMOTO, T. (1985). A v-erbB-related proto-oncogene, c-erbB-2, is distinct from the c-erbB-1/epidermal growth factor-receptor gene and is amplified in a human salivary gland adenocarcinoma. *Proc. Natl Acad. Sci.*, **82**, 6497.

SLAMON, D.J., CLARK, G.M., WONG, S.G., LEVIN, W.J., ULLRICH, A. & McGUIRE, W.L. (1987). Human breast cancer: correlation of relapse and survival with amplification of the HER-2/neu oncogene. *Science*, **235**, 177.

SMITH, C.H., NELSON, D.M., KING, B.F. & 3 others (1977). Characterization of a microvillous membrane preparation from placental syncytiotrophoblasts: a morphological, biochemical and physiological study. *Am. J. Obstet. Gynecol.*, **128**, 190.

SORGE, L.K., LEVY, B.T. & MANESS, P.F. (1984). pp60$^{c\text{-}src}$ is developmentally regulated in the neural retina. *Cell*, **36**, 249.

TAL, M., KING, C.R., KRAUS, M.H., ULLRICH, A. & 3 others (1987). Human HER2 (neu) promoter: evidence for multiple mechanisms for transcriptional initiation. *Molec. Cell. Biol.*, **7**, 2597.

TAL, M., WETZLER, Z., JOSEFBERG, A. & 7 others (1988). Sporadic amplification of the HER2/neu proto-oncogene in adenocarcinomas of various tissues. *Cancer Res.*, **48**, 1517.

TWARDZIK, D.R. (1985). Differential expression of transforming growth factor-$\alpha$ during prenatal development of the mouse. *Cancer Res.*, **45**, 5413.

VAN DE VIJVER, M., VAN DE BERSSELAAR, R., DEVILEE, P. & 3 others (1987). Amplification of the neu (c-erbB-2) oncogene in human mammary tumours is relatively frequent and is often accompanied by amplification of the linked c-erbA oncogene. *Molec. Cell. Biol.*, **7**, 2019.

VAN DE VIJVER, M.J., MOOI, W.J., WISMAN, P., PETERSE, J.L. & NUSSE, R. (1988). Immunohistochemical detection of the neu protein in tissue sections of human breast tumours with amplified neu DNA. *Oncogene*, **2**, 175.

VENTER, D.J., TUZI, N.L., KUMAR, S. & GULLICK, W.J. (1987). Overexpression of the c-erbB-2 oncoprotein in human breast carcinomas – immunohistological assessment correlates with gene amplification. *Lancet*, **ii**, 69.

WILCOX, J.N. & DERYNCK, R. (1988). Development expression of transforming growth factors alpha and beta in mouse fetus. *Molec. Cell. Biol.*, **8**, 3415.

WRBA, F., GULLICK, W.J., FERTL, H., AMANN, G. & SALZER-KUNTSCHIK, M. (1989). Immunohistochemical detection of the c-erbB-2 proto-oncogene product in normal, benign and malignant neoplastic human cartilage tissues. *Histopathology* (in the press).

YOTOTA, J., YAMAMOTO, T., TOYOSHIMA, K. & 4 others (1986). Amplification of c-erbB-2 oncogene in human adenocarcinomas in vivo. *Lancet*, **i**, 765.

YOKOTA, J., YAMAMOTO, T., MIYAJIMA, N. & 6 others (1988). Genetic alterations of the c-erbB-2 oncogene occur frequently in tubular adenocarcinoma of the stomach and are often accompanied by amplification of the v-erb-A homologue. *Oncogene*, **2**, 283.

ZHOU, D., BATTIFORA, H., YOKOTA, J., YAMAMOTO, T. & CLINE, M.J. (1987). Association of multiple copies of the c-erbB-2 oncogene with spread of breast cancer. *Cancer Res.*, **47**, 6123.

ZIMMERMAN, K.A., YANCOPOULOS, G.D., COLLUM, R.G. & 9 others (1986). Differential expression of myc family genes during murine development. *Nature*, **319**, 780.